# Clinical, Laboratory, and Imaging Features Associated with Arginine Vasopressin Deficiency (Central Diabetes Insipidus) in Erdheim–Chester Disease (ECD)

**DOI:** 10.3390/cancers17050824

**Published:** 2025-02-27

**Authors:** Sonal Vaid, Juvianee Estrada-Veras, William A. Gahl, Nicholas Patronas, Rahul H. Dave, Fady Hannah-Shmouni, Kevin O’Brien, Skand Shekhar

**Affiliations:** 1 Clinical Research Branch, National Institute of Environmental Health Sciences, National Institutes of Health (NIH), Research Triangle Park, NC 27709, USA; sonal.vaid@nih.gov; 2 National Institute of Diabetes and Digestive and Kidney Diseases, National Institutes of Health (NIH), Bethesda, MD 20892, USA; fshmouni@gmail.com; 3 National Human Genome Research Institute, National Institutes of Health (NIH), Bethesda, MD 20892, USA; juvianee.i.estradaveras.ctr@mail.mil (J.E.-V.); gahlw@mail.nih.gov (W.A.G.); 4 Clinical Center, National Institutes of Health (NIH), Bethesda, MD 20892, USA; njpatronas@gmail.com; 5 Inova Multiple Sclerosis and Neuro-Immunology Center, Fairfax, VA 22031, USA; rahul.dave@inova.org; 6 National Institute of Neurological Disorders and Stroke, National Institutes of Health (NIH), Bethesda, MD 20892, USA; 7 Department of Medicine, Division of Endocrinology, University of British Columbia, Vancouver, BC V5Z 1M9, Canada

**Keywords:** Erdheim–Chester disease, pituitary, central diabetes insipidus, arginine vasopressin deficiency, *BRAF* V600E

## Abstract

Purpose: Erdheim–Chester disease (ECD) is an L Group Langerhans histiocytosis associated with pathogenic variants within the MAPK pathways, most commonly the *BRAF* gene. We analyzed prevalence, genetic, biochemical, and pituitary imaging features associated with arginine vasopressin deficiency (AVP-D), one of the most common endocrinopathies in ECD. Methods: A cross-sectional descriptive study of 61 subjects with ECD was conducted at a clinical research center from January 2011 to December 2018, with molecular genetics, baseline biochemical and pituitary endocrine function studies, and dedicated pituitary MRI (or CT) studies. AVP-D and anterior pituitary endocrinopathies (hypothyroidism, hypogonadism, adrenal insufficiency and panhypopituitarism) were assessed. Students’ *t*-tests, nonparametric tests, Fisher’s exact tests, and logistic regression were employed for analysis. Results: In total, 22 out of 61 subjects (36%; 19 males and 3 females) had AVP-D; 18 subjects with AVP-D were in active treatment with desmopressin. Those with versus without AVP-D were younger [mean (±SD): 50.00 (±10.45) vs. 56.72 (±10.45) years], had higher prevalence of *BRAF* V600E pathogenic variants [68% vs. 43%], lower IGF-1 [mean (±SD): 137.05 (±67.97) vs. 175.92 (±61.89) ng/mL], lower urine osmolality [416.00 (250.00–690.00) vs. 644.50 (538.75–757.75)) mOsm/kg], and a higher burden of central hypogonadism [81.82% vs. 36.00%], central hypothyroidism [23% vs. 2.5%], panhypopituitarism [41% vs. 0%], anterior pituitary endocrine deficits, absent posterior pituitary bright spots [63.64% vs. 20.51%], and abnormal pituitary imaging. In adjusted models, [OR (95%CI)] *BRAF* V600E mutation [7.38 (1.84–39.01)], central hypogonadism [6.193 (1.44–34.80)], primary hypothyroidism [13.89 (1.401–406.5)], absent posterior pituitary bright spot [12.84 (3.275–65.04)], and abnormal pituitary imaging [10.60 (2.844–48.29)] were associated with higher odds of having AVP-D. Conclusions: AVP-D is common in ECD and accompanied by a higher burden of pituitary endocrinopathies, *BRAF* V600E pathogenic variants, abnormal pituitary imaging, and absent posterior pituitary bright spots.

## Simple Summary

Arginine vasopressin deficiency (AVP-D, previously known as central diabetes insipidus) is among the most common initial presentations of ECD, implying that a deeper understanding of AVP-D-related factors may help improve outcomes. We performed a cross-sectional analysis of clinical, molecular and imaging features associated with AVP-D in ECD. Subjects with AVP-D were younger, and had more central endocrine deficiencies and pituitary imaging abnormalities. Importantly, AVP-D was associated with a higher burden of *BRAF* V600E pathogenic variants, which is also a therapeutic target. Together, our findings provide insights for triaging the care of AVP-D in ECD, suggesting that subjects with AVP-D may benefit from a comprehensive hormonal, molecular, and radiological assessment.

## 1. Introduction

Erdheim–Chester disease (ECD) is a rare Group L Langerhans cell histiocytosis classified as a hematopoietic neoplasm by the World Health Organization [1]. Approximately 800 cases have been reported; however, the exact incidence remains unknown [2]. ECD commonly manifests as an insidious multisystemic disorder warranting treatment. In rare cases, it may appear as a minimally symptomatic condition involving a single system and may not require treatment [2]. Somatic pathogenic variants in the MAP (mitogen-activated protein) kinase pathway, like deleterious pathogenic variants in *NRAS, KRAS, ARAF, MAP2K1, ALK*, or gain-of-function mutations in *BRAF* V600E, have most commonly been reported; however, mutations in *PIK3CA* have also been noted [3,4]. Histopathologic findings often consist of nonspecific inflammation, fibrosis, and tissue infiltration by CD68+, CD1a-, xanthogranulomatous, and lipid-rich foamy macrophages [5].

The most common clinical manifestations of ECD include neurological symptoms, bone disease, arginine vasopressin deficiency (AVP-D, formerly known as central diabetes insipidus), and associated constitutional symptoms [6]. The skin, retroperitoneal, cardiovascular, and pulmonary systems are other commonly affected organ systems [6]. The clinical course of ECD depends upon the severity of organ involvement and may change over time. Before the advent of targeted molecular therapy, treatment of ECD relied on glucocorticoids, recombinant human interleukin-1 receptor antagonist (IL-1RA), cladribine, interferon alpha [interferon-α (IFN-α)], and tyrosine kinase inhibitors. The advent of *BRAF* V600E inhibitors and MEK (mitogen-activated protein kinase) inhibitors has transformed the care of patients with ECD [3,6,7]. Asymptomatic patients without organ dysfunction can be serially monitored both clinically and radiologically, without directed therapy [8].

Importantly, one of the most common and frequently overlooked endocrinopathies in ECD is AVP-D, with a median diagnostic delay from the first symptom of 5 years [6]. In our study population, the mean time of diagnostic delay, defined as the time lapsed between the appearance of the first symptoms of ECD and its formal diagnosis, was 4.2 years [9]. AVP-D due to a deficiency in AVP (arginine vasopressin) is characterized by hypotonic, dilute polyuria (low urine osmolality), polydipsia, elevated plasma osmolality, and sometimes hypernatremia [10]. Given the frequency of misdiagnoses and high burden of AVP-D in ECD [6], it is important to understand associations between AVP-D status and clinical, biochemical, genetic, and radiological features that could guide management approaches in patients with AVP-D in ECD. In order to describe the nature, frequency, and factors associated with AVP-D in patients affected by ECD, we conducted a cross-sectional study on a cohort of patients with ECD.

## 2. Materials and Methods

### 2.1. Overview

Subjects with ECD were enrolled in a National Institutes of Health (NIH) IRB-approved natural history observational study “Clinical and Basic Investigations into Erdheim–Chester disease” (Protocol 11-HG-0207, ClinicalTrials.gov Identifier: NCT01417520) and gave written, informed consent. Between January 2011 and December 2018, subjects were evaluated at the National Institutes of Health Clinical Center, and the diagnosis was established based on ECD consensus criteria [2,8].

### 2.2. Procedures

A cross-sectional analysis of clinical, biochemical, and radiological characteristics was performed. Diagnoses were verified with history, physical examination and laboratory, genetic, and imaging investigations such as dedicated pituitary MRI in 56 subjects and CT scans of the sella in 5 subjects. MRIs were reviewed by a neuroradiologist and confirmed by neuroendocrinologists (FHS, SS) with expertise in pituitary disorders. A certified pathologist reviewed biopsy samples (common sites, perinephric or retroperitoneal tissue, bone, and skin) and confirmed the diagnosis of ECD by excluding other diagnoses such as IgG4-related diseases. A diagnosis of endocrinopathies based on well-established criteria was ascertained after a detailed review of historical records, medical history and examination, and laboratory assessment at NIH. Screening for the *BRAF* V600E pathogenic variant was conducted via molecular genetic testing using the polymerase chain reaction technique, with sequencing for exons 11 and 15, among 25 subjects. Genetic reports were not available for four subjects (two with and two without DI). Subjects testing negative for *BRAF* V600E underwent testing for MAPK genes in *MAP2K1*, *PIK3CA*, *KRAS*, *NRAS*, and *ARAF* via dideoxy sequencing. Data for each subject were collected at the baseline (enrollment) visit.

Our primary objective was to determine the frequencies of clinical, laboratory, and imaging features associated with AVP-D. Our secondary objective was to analyze the following variables in ECD subjects with and without AVP-D: age, body mass index (BMI), sex, high-sensitivity C-reactive protein (hsCRP) levels, *BRAF* V600E status, anterior pituitary hormonal deficits, and abnormal pituitary imaging. Biochemical evaluation included serum sodium (mmol/L), serum osmolality (mOsm/kg), urine osmolality (mOsm/kg), serum arginine vasopressin (pg/mL), plasma hsCRP (mg/dL), plasma 8 AM total testosterone (ng/dL), serum prolactin (mcg/L), serum IGF-1 (insulin-like growth factor-1, ng/mL), and *BRAF* V600E pathogenic variant status. At enrollment, some subjects were undergoing treatment with BRAF inhibitors, interferon therapy, or corticosteroids or were receiving other forms of treatment [11]. Reported laboratory and radiological data were collected as part of the study at NIH.

### 2.3. Radiological Assessment

In total, 56 subjects underwent magnetic resonance imaging (MRI) of the pituitary on a 3.0 Tesla Philips Achieva^®^ using T1- and T2-weighted imaging with sagittal, coronal, and axial sequences; 5 subjects underwent CT scans of the sella with axial images obtained at 2 mm intervals. Unenhanced and contrast-enhanced images were obtained before and after an intravenous injection of Magnevist^®^ (gadopentetate dimeglumine) and (Isovue) contrast as described previously [11]. Imaging characteristics and abnormalities of imaging were determined according to standardized criteria [11]. We specifically assessed images for pituitary stalk thickening and/or deviation, partially empty sella, pituitary atrophy, abnormal pituitary enhancement, Rathke’s and pars intermedia cysts, and loss of posterior pituitary bright spots. If this imaging finding was considered secondary to ECD, it was labeled as abnormal pituitary imaging (API) [11].

### 2.4. Endocrine Assessment

Medical records were reviewed for all enrolled subjects. We considered subjects to have AVP-D if subjects met the criteria for AVP-D after (1) undergoing an outpatient evaluation (plasma and urine osmolality evaluation, water deprivation tests, etc.) in the setting of a suggestive clinical history (e.g., polyuria, polydipsia), prior to or during their NIH assessment, or (2) were treated with desmopressin at the time of or any timepoint preceding enrollment at NIH. Urinary samples were collected in the morning, and subjects were euvolemic (18 subjects were on DDAVP at the time of testing). Four subjects were not on DDAVP, because upon their arrival at the NIH, they had undergone spontaneous remission and were not actively requiring any DDAVP despite previously being on it. Central hypothyroidism, central hypogonadism, central adrenal insufficiency, and panhypopituitarism (≥3 anterior pituitary deficits) were determined according to previously described standardized criteria [11,12]. Dynamic testing for growth hormone deficiency was not performed, and thus, GH deficiency did not contribute to diagnosing panhypopituitarism. We have previously reported details on the burden and nature of hypothyroidism and hypothalamic–pituitary–adrenal involvement in our study population [13].

### 2.5. Statistical Analysis

Data are reported as frequencies, percentages, means and standard deviations (SD), or medians and interquartile ranges (IQR). Categorical data such as the frequency of characteristics were compared using Fisher’s exact tests by constructing 2 × 2 tables, and continuous data were analyzed using *t*-tests or nonparametric tests to assess differences between those with and without AVP-D. Multivariate regression analysis was performed to confirm independent associations based on signals of association from univariate analysis. All analyses were conducted using available data and by excluding missing values.

## 3. Results

A total of 61 subjects with ECD were enrolled in the protocol and included in the final analysis [15 (25%) females and 46 (75%) males]. A total of twenty-two subjects (36%) had AVP-D (86% males versus 14% females) (Table 1). For all subjects, the age at presentation ranged from 16 to 74 years (mean, 54 years), and the mean time to diagnosis was 4.2 years. Fifteen subjects (~25% of those with ECD) had AVP-D among the initial manifestations of ECD with a mean age (SD) at presentation of 37.53 ± 10.18 years, a mean age at diagnosis of 47.40 ± 9.67 years, and a mean diagnostic delay from first symptom of 9.87 years.

**Figure 1:**
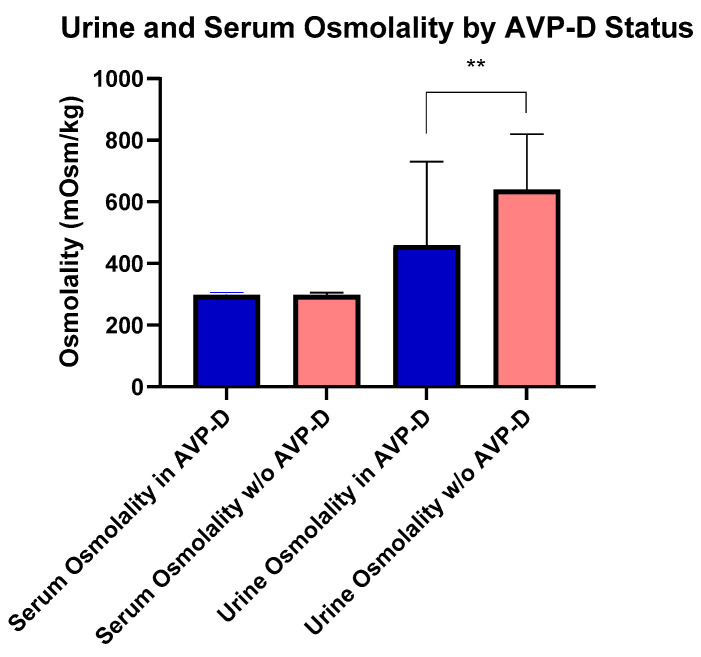
Comparisons of urine and serum osmolality between those with and without AVP-D. Urine osmolality was higher in those without AVP-D compared to those with AVP-D as denoted by double asterisks (*p* < 0.01).

At the time of their NIH evaluation, four subjects with AVP-D were following behavioral strategies to maintain fluid balance (e.g., increased oral intake) and not receiving DDAVP due to the mild nature of their AVP-D. Subjects with AVP-D were younger (54.30 ± 10.93 years), but had a similar BMI compared to those without AVP-D. Subjects with DI had lower IGF-1 (161.67 ± 67.43 ng/mL) than those without AVP-D (Table 1). Importantly, serum and urine osmolality were not different among subjects with DI on and off of DDAVP, respectively (*p* = 0.9 and *p* = 0.4). There were no differences in serum vasopressin, serum osmolality, serum sodium, or BMI (Table 1) between those with and without AVP-D even as 18 subjects with AVP-D were receiving desmopressin. Fifteen subjects were receiving long-term glucocorticoids, out of whom six subjects were on supraphysiological glucocorticoid dosing for ECD. Among the remaining subjects, seven met the criteria for central adrenal insufficiency and two for primary adrenal insufficiency [14,15], without differences in body surface area (BSA)-based daily replacement dosing [median (range) in 14.5 (12.81–14.85) vs. 16.98 (14.85–14.85) mg/m^2^ BSA/day, *p* = 0.33]. In those not on exogenous glucocorticoids, serum morning cortisol levels were similar between subjects with and without AVP-D (*p* = 0.21). Similarly, for those subjects who had central or primary hypothyroidism, weight-based levothyroxine [mean (range) LT4 dose in mcg/kg body weight per day, central vs. primary dose: 1.54 (1.5–1.61) vs. 1.26 (1.05–1.80), *p* = 0.43] was similar. Urine osmolality was lower in those with AVP-D vs. without AVP-D [416.00 (250.00–690.00) mOsms/kg (n = 18)] vs. [644.50 (538.75–757.75) mOsms/kg (n = 39)] (Figure 1). In subjects with AVP-D, central hypothyroidism [5 (23%) vs. 1 (2.5%)], central hypogonadism [18 (82%) vs. 14 (36%)], and panhypopitutiarism [9 (41%) vs. 0 (0%)] were more common than in those without AVP-D. While dynamic testing for GH deficiency was not performed in our study subjects, we found low IGF-1 levels (age and sex matched) in two subjects. Both subjects had AVP-D and one had panhypopituitarism. Among the 15 women in the cohort, 9 were postmenopausal. Gonadotropin deficiency, as defined by menopause status reference ranges, was identified in five women. Among these five, three had AVP-D, all of whom were postmenopausal. No differences were noted in total cholesterol, hsCRP, estradiol, total testosterone in males, cortisol, TSH, free T4, or prolactin between groups (Table 1).

Overall, 57 subjects were tested for pathogenic variants in the *BRAF* V600E gene, and 31 were noted to harbor a somatic pathogenic variant in *BRAF* V600E. Among those with a wild-type BRAF V600E, 12 had additional testing for genetic pathogenic variants. Two subjects, both without AVP-D, were noted to have additional pathogenic variants, with one having a pathogenic variant in *ARAF* D228V and the other an activating *ALK* fusion (KIF5B-ALK). Notably, subjects with AVP-D had a higher burden of *BRAF* V600E pathogenic variants (68% vs. 43%) (Table 1).

**Figure 2:**
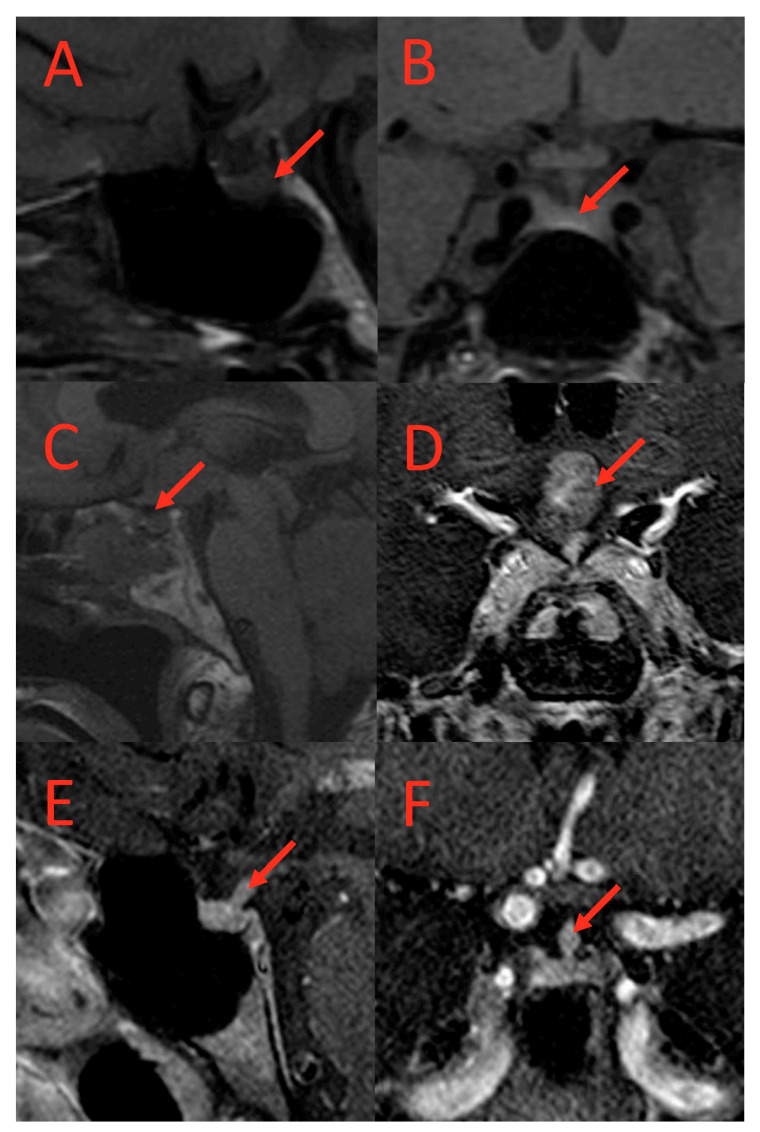
T1-weighted MRI scans of subjects with AVP-D. (**A**) (coronal) and (**B**) (sagittal): Loss of posterior pituitary bright spot in a subject with AVP-D. (**C**,**D**): Suprasellar mass in a subject with DI. (**E**,**F**): Thickened pituitary stalk at hypothalamus in a subject with AVP-D and loss of posterior pituitary bright spot. Red arrows point to the lesion.

Similarly, abnormal pituitary imaging (81.82% vs. 28.21%) and absent posterior pituitary bright spots on MRI (64%% vs. 21%) were more common in those with AVP-D compared to those without AVP-D (Table 1 and Table 2). The frequencies of small pituitary, thickened pituitary stalk, stalk deviation, pituitary encasement, complete empty sella, partial empty sella, pars intermedia cyst, Ratkhe’s cyst, microadenoma, absent posterior lobe, and suprasellar mass were similar between the two groups (Table 2). Representative images of pituitary imaging abnormalities associated with AVP-D are shown in Figure 2.

On multivariate regression analysis, with covariates of age, sex, and BMI, the male sex [Odds Ratio (95%CI) 5.222 (1.172 to 33.89)] was associated with higher odds of AVP-D, and advancing age (beta ± standard error: −0.08 ± 0.03) was inversely associated with AVP-D. In models adjusting for age, sex, and BMI, the *BRAF* V600 E pathogenic variant [OR (95%CI) 7.38 (1.84 to 39.01)], central hypogonadism [OR (95%CI) 6.19 (1.44 to 34.80)], and primary hypothyroidism [OR (95%CI) 13.89 (1.40 to 406.5)] emerged as being associated with a higher odds of having AVP-D (Table 3). Panhypopituitarism and the number of pituitary deficits were excluded from the analysis due to collinearity. After similar adjustments, IGF-1 and urine osmolality were inversely associated, and total testosterone was positively associated with AVP-D status, respectively (Table 3). Imaging features such as absent posterior pituitary bright spots [OR (95%CI) 12.84 (3.275 to 65.04)] and abnormal pituitary imaging [OR (95%CI) 10.60 (2.844 to 42.89)] were also independently associated with higher odds of DI in models adjusting for age, sex, and BMI (Table 2 and Table 3).

## 4. Discussion

Our cross-sectional, descriptive natural history study cohort of 61 ECD subjects revealed that AVP-D was present in 36% of subjects. Univariate analysis revealed that subjects with AVP-D were younger and had a higher burden of *BRAF* V600E pathogenic variants and clinical and biochemical endocrine deficiencies compared to those without AVP-D, such as a higher prevalence of panhypopituitarism, central hypogonadism, and central hypothyroidism and lower urine osmolality and serum IGF-1 levels. Multivariate analysis adjusting for age, BMI, and sex demonstrated that younger age, male sex, *BRAF* V600E pathogenic variants, central hypogonadism, and primary hypothyroidism were independently associated with a higher odds of AVP-D. There were inverse associations between AVP-D status and serum IGF-1 and urine osmolality. These findings confirm and extend previously published reports on the burden of AVP-D in ECD while contributing additional insights into differences in the phenotype between those with and without AVP-D.

Physiologically, vasopressin is secreted by the posterior pituitary gland and then acts on the renal collecting ducts to increase free water reabsorption and maintain plasma tonicity [16,17]. AVP-D in histiocytoses is thought to occur through lymphocytic infiltration and the destruction of the hypothalamic–neurohypophysial unit; however, there may be other contributing mechanisms that have not yet been fully defined. AVP-D is among the earliest presenting features of ECD and may be accompanied by years of misdiagnosis and diagnostic delays that result in lost therapeutic opportunity [6,9]. Such delays in diagnosis may occur because those with an intact thirst response are able to compensate for urinary-free water losses by increased intake of fluids, resulting in nonspecific polyuria and polydipsia, which may not be bothersome enough for patients to seek medical attention [18]. Furthermore, clinical recognition of AVP-D may be challenging in inexperienced hands due to a broad differential diagnosis (e.g., psychogenic polydipsia) and complex etiologies. Additionally, patients with AVP-D do not develop symptoms other than polyuria and polydipsia (e.g., dehydration, nocturia, etc.) until their hypothalamic secretion of AVP drops below 15%, which occurs relatively late in the disease course [18]. The complex task of linking an elusive feature, such as AVP-D, to an uncommon etiology, such as ECD, contributes to misdiagnoses and diagnostic delays. In our study, the frequency of AVP-D (36%) was comparable but somewhat higher than previous estimates (22–33%), which may reflect differences in study designs [6,19]. ECD is primarily a disease of middle-aged adults, with a mean age of 46–56 years [2], consistent with our cohort’s mean age. Interestingly, our subjects with AVP-D were younger in comparison to those without AVP-D, and multivariate regression confirmed an inverse association between advancing age and predisposition to developing AVP-D. This is consistent with a report by Cavalli et al., who found a higher prevalence of AVP-D in younger ECD patients [29% (age < 40 y) vs. 20% (age 40–70 y)] [6]; however, the precise nature and extent of these age-related differences in AVP-D warrant further elucidation.

We also found that AVP-D patients had a lower urine osmolality than those without AVP-D despite the majority of them being treated with DDAVP. Differences in osmolality would have been expected to be larger if patients had not been treated with DDAVP. This may reflect incomplete renal medullary gradient correction in the presence of exogenous DDVAP, even though serum sodium, vasopressin, and osmolarity returned to the normal range in subjects with AVP-D [10]. Since many patients were on vasopressin replacement treatment, it is likely that our vasopressin assay could not actually detect the difference between subjects who were treated and those who were not, which may have been overcome if copeptin levels were measured. Moreover, the four subjects who had a history of AVP-D but did not receive vasopressin replacement upon NIH enrollment suggest a fluctuating infiltrative course of ECD that leads to AVP-D.

Endocrinopathies are common in patients with ECD as we have previously reported [9]. For instance, we noted that central and primary hypothyroidism was present in 9.8% and 18% of ECD patients, respectively [12]. Other endocrinopathies such as GH deficiency, central hypogonadism, central adrenal insufficiency, and panhypopituitarism are also common in individuals with ECD [11,13,19]. In the current study, we found a higher burden of central hypogonadism, central hypothyroidism, and panhypopituitarism, which signals a higher burden of central neuroendocrine disruption. Such signals were confirmed in multivariate regression analysis, where central hypogonadism continued to be associated with AVP-D, with six-fold odds of AVP-D in those with central hypogonadism. Notably, serum IGF-1, a marker of GH secretion, was inversely associated with AVP-D, supporting the hypothesis of significant anterior pituitary dysfunction in patients with AVP-D, although the underlying mechanisms remain unclear. Despite hsCRP, an inflammatory marker, not varying by AVP-D status, the plausible link between a higher inflammatory burden and AVP-D warrants further investigation since autoimmune destruction (primary hypothyroidism) was also associated with AVP-D in multivariate regression. Importantly, hyperprolactinemia did not vary based on AVP-D status, which may reflect significant pituitary stalk compression or impingement. This is likely explained by pituitary stalk infiltration or hypothalamic destruction, resulting in AVP-D being insufficient to result in hyperprolactinemia, as corroborated by frequencies of stalk thickening and deviations being similar between those with and without AVP-D (Table 2). It is also possible that a rise in prolactin is insidious, and hyperprolactinemia may develop over time, an outcome that our cross-sectional study could not capture.

Upon multivariate analysis, *BRAF* V600E pathogenic variants carried a seven-fold higher odds of having AVP-D, indicating an increased risk of developing AVP-D in those carrying this pathogenic variant; however, this needs confirmation in future studies [20]. This link also carries importance given the advent of *BRAF* V600E-targeted therapy (vemurafenib and dabrafenib), which may be particularly valuable in those with AVP-D. An important unanswered question is whether the early initiation of such therapy could attenuate or reverse the course of AVP-D in affected patients. Regardless, molecular testing for *BRAF* V600E and MAPK pathway defects should be prioritized in ECD patients with AVP-D.

Posterior pituitary bright spots are found in 90–100% of healthy people on T1-weighted MRI scans and reflect the physiological storage of vasopressin crystals [21]. Univariate followed by multivariate analysis demonstrated higher odds of AVP-D in those with absent posterior pituitary bright spots (~13-fold increase odds) and abnormal pituitary imaging (~11-fold increase odds), further substantiating a pituitary clinical–radiological association in ECD patients with AVP-D, with important implications for the clinical management of ECD patients with AVP-D.

The current study has several strengths. This is amongst the largest cohorts of patients with ECD systematically evaluated and phenotyped. All our subjects had extensive history and physical examinations, biochemical testing, histopathological confirmation of the diagnosis and relevant radiological assessment performed. We also performed molecular genetic testing on our subjects.

Our study has some limitations. Since this was a cross-sectional analysis, we could not determine the course of the disease over time or assess its incidence. In some cases, we had to rely on previously performed workup for AVP-D, which was not always performed during their study visit. Even though our study consisted of a large cohort of ECD patients reflecting the natural prevalence of the disease, our study was not powered to investigate sex-related differences. Thus, male sex as a risk factor for AVP-D must be confirmed in follow-up studies. Moreover, GH deficiency was not included in our definition of panhypopituitarism, given the lack of provocative testing, which may have underestimated the burden of panhypopituitarism. We also did not measure serum copeptin, which is a stable surrogate marker for endogenous AVP concentrations, which would be useful in quantifying the (deficient) secretion of AVP in future studies. Furthermore, some subjects were on supraphysiological GC replacement, which may have influenced test results. Additionally, we were not able to assess the impact of pharmacotherapy upon AVP-D or ECD-related outcome measures.

Our study provides clinicians with critical insights into the clinical, biochemical, genetic, and imaging profiles of ECD patients with AVP-D. AVP-D was noted in a significant proportion of patients with ECD accompanied by a large burden of other endocrinopathies, more hypotonic urine despite vasopressin therapy, the absence of posterior pituitary bright spots, and pathogenic variants in *BRAF* V600E compared to those without AVP-D. Collectively, these findings suggest that those with ECD and co-existing AVP-D may benefit from a comprehensive assessment of endocrine dysfunction consisting of biochemical and radiological evaluations, prioritized testing for *BRAF* V600E pathogenic variants, and close monitoring of fluid balance.

## 5. Conclusions

In conclusion, arginine vasopressin deficiency is widely prevalent in ECD, which is associated with a younger age, a higher frequency of *BRAF* V600E pathogenic variants, anterior pituitary endocrine dysfunction, absent T1-posterior pituitary bright spots, and abnormal pituitary imaging. The advent of molecular therapy targeting *BRAF* V600E and related pathways presents an opportunity for future studies to determine the impact of such therapies on AVP-D.

## Figures and Tables

**Table 1:** Clinical characteristics and laboratory values of subjects with and without arginine vasopressin deficiency (AVP-D) in Erdheim–Chester disease (ECD). *p* values listed for AVP-D vs. non-AVP-D subjects.

Characteristics: [Reference Ranges]	Full Cohort (n = 61)	Subjects with AVP-D (n = 22)	Subjects Without AVP-D (n = 39)	*p* Value
Age, mean (SD), y	54.30 ± 10.93	50.00 ± 10.45	56.72 ± 10.45	<0.01
Sex, female vs. male, n (%)	15 (24.59%) vs. 46 (75.40%)	3 (13.63%) vs. 19 (86.36%)	12 (30.77%) vs. 27 (69.23%)	0.99
Body Mass Index, median (IQR), kg/m^2^	27.80 (24.80–32.90)	29.65 (24.50–34.50)	27.20 (25.20–32.45)	0.82
Serum Sodium, median (IQR), mmol/L [135–144 mmol/L]	141 (140.00–143.00)	141(140.00–142.75)	141 (139.50–143.00)	0.94
Serum Osmolality, median (IQR), mOsm/kg [278–298 mOsm/kg]	299 (295.00–302.00)	300 (296.00–302.00)	297 (294.50–302.00)	0.37
Urine Osmolality, median (IQR), mOsm/kg (n = 57) [300–900 mOsm/kg]	604.00 (403.00–721.00)	416.00 (250.00–690.00)	644.50 (538.75–757.75)	<0.01
Arginine Vasopressin, median (IQR), pg/mL [less than 1.7 pg/mL]	0.70 (0.25–1.20)	0.48 (0.25–1.20)	0.70 (0.25–1.20)	0.7
Total Cholesterol, mean (SEM), mg/dL [less than 200 mg/dL]	167.59 ± 4.52	173.91 ± 6.91	164.03 ± 5.89	0.29
hsCRP, median (IQR), mg/dL [less than 1: low risk 1–3: average risk Greater than 3 high risk]	13.40 (4.06–48.65)	19.80 (7.11–63.60)	6.64 (2.73–45.05)	0.12
Total Testosterone in males, median (IQR), ng/dL [262–1593 ng/dL]	266.00 (177.50–414.50) (n = 46)	382.00 (194.25–567.50) (n = 18)	246.50 (156.50–355.00) (n = 28)	0.07
Estradiol in females, median (IQR), pg/mL [12–460 pg/mL]	7.75 (5.50–26.10) (n = 13)	6.30 (5.60–23.00) (n = 5)	10.40 (5.38–26.30) (n = 8)	0.80
Prolactin, median (IQR) mcg/L [2–25 mcg/L]	8.90 (6.40–15.60)	8.55 (6.25–15.05)	9.40 (6.70–14.00)	0.81
IGF-1, mean (SEM), ng/mL [94–252 ng/mL]	161.67 ± 8.71	137.05 ± 13.87	175.92 ± 10.22	0.03
TSH, median (IQR), (mcIU/mL) [0.40–4.00 mcIU/mL]	1.68 (0.94–2.77)	1.58 (0.54–2.47)	1.57 (0.95–2.46)	0.48
Free T4, median (IQR), (ng/dL) [0.8–1.5 ng/dL]	1.20 (1.10–1.30) (n = 23)	1.30 (1.20–1.50) (n = 9)	1.20 (1.18–1.20) (n = 14)	0.13
Serum Cortisol, mean (SEM), (mcg/dL) [5–25 mcg/dL] *	11.90 ± 0.86	10.12 ± 1.45	15.13 ± 1.05	0.12
Pathogenic Variant: *BRAF* V600 E, n (%)	31 (50.81)	15 (88.23) (n = 17)	16 (43.24) (n = 37)	<0.01
Panhypopituitarism, n (%)	9 (14.75)	9 (40.90)	0 (0.00)	<0.01
Central Hypogonadism, n (%)	32 (52.45)	18 (81.82)	14 (36.00)	<0.01
Central Hypothyroidism, n (%)	6 (9.83)	5 (22.73)	1 (2.56)	0.02
Primary Hypothyroidism, n (%)	11 (18.03)	3 (13.63)	8 (20.51)	0.73
Central Adrenal Insufficiency, n (%)	7 (11.48)	5 (22.73)	2 (5.13)	0.09

For those variables where one or more values were missing, total n values are listed separately in parenthesis. Abbreviations: hsCRP, high-sensitivity C-reactive protein; SD, standard deviation; SEM, Standard Error of the Mean; IQR, interquartile range; IGF-1, insulin-like growth factor-1. * Values only reported for those not on exogenous glucocorticoids.

**Table 2:** Comparison of imaging features in subjects with and without arginine vasopressin deficiency (AVP-D) in Erdheim–Chester disease (ECD). *p* values listed for comparisons between those with and without AVP-D.

Characteristics	Full Cohort (n = 61)	Subjects with AVP-D (n = 22)	Subjects Without AVP-D (n = 39)	*p* Value
Absent Posterior Pituitary Bright Spot, n (%)	22 (36.07)	14 (63.64)	8(20.51)	<0.01
Abnormal Morphology–Heterogenous Enhancement, n (%)	11 (18.03)	6 (27.27)	5 (12.82)	0.3
Small Pituitary, n (%)	9 (14.75)	5 (22.73)	4 (10.26)	0.26
Thickened Pituitary Stalk, n (%)	15 (24.59)	7 (31.82)	8 (20.51)	0.36
Stalk Deviation, n (%)	7 (11.48)	2 (9.09)	5 (12.82)	0.99
Pituitary Encasement, n (%)	3 (4.92)	1 (4.55)	2 (5.13)	0.99
Complete Empty Sella, n (%)	4 (6.56)	1 (4.55)	3 (7.69)	0.99
Partial Empty Sella, n (%)	4 (6.56)	1 (4.55)	3 (7.69)	0.99
Intermediate Cyst in Sella, n (%)	3 (4.92)	1 (4.55)	2 (5.13)	0.99
Ratkhes Cyst in Sella, n (%)	1 (1.64)	0 (0.00)	1 (2.56)	0.99
Microadenoma, n (%)	1 (1.64)	1 (4.55)	0 (0.00)	0.36
Absent Posterior Lobe, n (%)	2 (3.28)	1 (4.55)	1 (2.56)	0.99
Suprasellar Mass, n (%)	2 (3.28)	2 (9.09)	0 (0.00)	0.13
Abnormal Pituitary Imaging, n (%)	29 (47.54)	18 (81.82)	11 (28.21)	<0.01

**Table 3:** Multivariate regression analysis.

Continuous Variable	Beta Estimate ± SE	95% CI
Age	−0.084 ± 0.031	−0.149 to −0.027
Insulin-Like Growth Factor	−0.012 ± 0.005	−0.024 to −0.002
Osmolality (Urine)	−0.004 ± 0.002	−0.007 to −0.001
Total Testosterone	0.003 ± 0.002	0.00045 to 0.001
**Categorical Variable**	**Odds Ratio (95% CI)**	
Male Sex	5.22 (1.17 to 33.89)	
*BRAF* V600E Pathogenic Variant	7.38 (1.84 to 39.01)	
Central Hypogonadism	6.19 (1.44 to 34.80)	
Primary Hypothyroidism	13.89 (1.40 to 406.50)	
Absent Pituitary Bright Spot	12.84 (3.28 to 65.04)	
Abnormal Pituitary Imaging	10.6 (2.84 to 48.29)	

All models were run using the following independent variables: sex (except for total testosterone which was only in males), age, body mass index, and an outcome variable of arginine vasopressin deficiency (AVP-D) (present vs. absent). CI: confidence interval; SE: standard error.

## Data Availability

Data are contained within the article. The remaining data presented in this study are available on request from the corresponding author.
